# The prognostic value and immunological role of MVP in pan-cancer study

**DOI:** 10.18632/aging.205802

**Published:** 2024-05-06

**Authors:** Chunlin Li, Min Gao, Nashunbayaer Zha, Gang Guo

**Affiliations:** 1 Department of Thoracic Surgery, The Affiliated Hospital of Inner Mongolia Medical University, Hohhot 010010, China; 2 Inner Mongolia Medical University, Hohhot 010010, China; 3 Department of Thyroid and Breast Surgery, The Affiliated Hospital of Inner Mongolia Medical University, Hohhot 010010, China

**Keywords:** MVP, pan-cancer, prognosis, methylation, immune

## Abstract

Major Vault Protein (MVP) has emerged as a potential prognostic and immunological biomarker in various cancer types. This pan-cancer study aimed to investigate expression of MVP and its correlation with clinical outcomes and immune infiltration across diverse cancer types. We conducted an analysis of extensive transcriptomic and clinical data from publicly available databases. Our findings unveiled a significant association between MVP expression and cancer progression, with higher expression levels predicting poorer overall survival in multiple cancer types. Importantly, MVP expression demonstrated a close relationship with immune infiltration in the tumor microenvironment, showing that higher expression levels were associated with increased immune cell infiltration. We further validated expression of MVP and function in cancer cell lines A549 and AGS. These compelling results suggest that MVP holds promise as a valuable biomarker for prognostic assessment and the development of immunotherapeutic strategies across various cancer types. Consequently, targeting MVP may offer a compelling therapeutic approach in the treatment of human cancers.

## INTRODUCTION

Cancer remains a formidable global health challenge despite significant advancements in early detection and treatment, which have contributed to a decline in cancer-related mortality rates [1]. However, the incidence and impact of cancer continue to rise, primarily due to population aging, resulting in persistently low five-year survival rates [2]. Therefore, it is imperative to explore innovative approaches for cancer diagnosis and treatment. The utilization of cancer biomarkers has shown promise, motivating researchers to investigate novel targets for cancer therapy and prognostic markers. Consequently, there is an urgent demand for innovative strategies to enhance cancer diagnosis and therapy, ultimately leading to improved patient outcomes.

Major Vault Protein (MVP) is the predominant constituent of the vault complex, which is the largest known ribonucleoprotein particle. The vault complex is hypothesized to serve as a versatile carrier molecule facilitating nucleocytoplasmic transport. With its distinctive barrel-shaped structure, MVP exhibits significant conservation across diverse species [3–6]. Notably, the involvement of MVP in chemotherapy resistance and its correlation with prognosis have been observed in several cancer types, such as triple-negative breast cancer [7], liver cancer [8], lung cancer [9], and colon cancer [10]. Recent investigations have revealed that the interaction of MVP with interferon regulatory factor 2, as well as its inhibition of p53 activity, may contribute to hepatocellular carcinoma development. Furthermore, elevated MVP expression has been associated with hepatocellular carcinoma progression and poorer overall survival (OS) [11]. Additionally, MVP has been implicated in fine-tuning inflammatory activation processes, including apoptosis signaling mediated by immuno-monitored cytokines like tumor necrosis factor-associated apoptosis-inducing ligands [12]. Moreover, MVP has demonstrated links to resistance-related signaling pathways such as PI3K/AKT and MAPK pathways [13, 14]. Given its pivotal role in tumorigenesis and chemoresistance, MVP emerges as a crucial regulator of diverse cellular processes. Further investigation is necessary to elucidate the mechanisms and functions of MVP, thus facilitating the identification of potential therapeutic targets for cancer treatment.

In this study, we conducted a comprehensive bioinformatics analysis utilizing multiple databases to explore the expression levels, genetic alterations, and prognostic significance of MVP across normal tissues and various cancer types. Furthermore, we assessed the association between MVP expression levels and DNA methylation, immune cell infiltration, and immune checkpoints. Additionally, we conducted functional experiments by depleting MVP in A549 and AGS, which led to attenuated cell proliferation, migration, invasion and promote apoptosis. Through these endeavors, we aim to offer novel perspectives on the involvement of MVP in the pathogenesis, therapeutic interventions, and prognostication of human tumors.

## MATERIALS AND METHODS

### Data collection and processing

An analysis of MVP expression was conducted in 34 tumors and their corresponding normal tissues by utilizing the TCGA dataset combined with the Genotype Tissue Expression (GTEx) cohort. The SangerBox (http://SangerBox.com/Tool) platform was employed for the analysis. To determine the subcellular localization of MVP, we accessed the online Human Protein Atlas (HPA) portal (https://www.proteinatlas.org/) and entered “MVP” in the “Subcellular” module. Additionally, the expression levels of MVP protein were examined using the Clinical Proteomic Tumor Analysis Consortium (CPTAC) module accessible through the UALCAN portal (http://ualcan.path.uab.edu/index.html) [15].

### Epigenetic methylation analysis

Differences in MVP-promoter methylation levels were investigated between 16 tumors and their corresponding normal tissues on the UALCAN portal. The database applies TPM normalization to the methylation expression values obtained from TCGA. Various beta cut-off points were employed to define low methylation (b: 0.3-0.25) and hypermethylation (b: 0.7-0.5) states [16, 17]. Additionally, the TIDE server [18] was employed to assess the impact of methylation on dysfunctional T cell phenotype and prognosis.

### Genetic alteration analysis

MVP gene alterations were examined through the utilization of the cBioPortal database (https://www.cbioportal.org/) [19, 20]. The TCGA Pan-Cancer Atlas Study dataset was queried using the keyword “MVP”, and the Cancer Type Summary module was utilized to determine the frequency and type of gene mutations and copy number variations.

### MVP-related genes enrichment analysis

In order to identify potential genes associated with MVP, we employed the STRING database. Specifically, we conducted a search for “MVP” in the Protein Names module and “Homo sapiens” in the Organisms module. From the TCGA dataset, we obtained the top 100 genes linked to MVP using GEPIA2’s “similarity gene detection” function. Subsequently, we employed “Correlation Analysis” of GEPIA2 module to examine the correlation between MVP and the top 5 genes [21]. Scatter plots were constructed based on log2 TPM data. To gain insights into the potential biological processes and pathways associated with the identified genes, we performed pathway and process enrichment analysis using the Metascape web-based tool. The selection of enriched canonical pathways was based on parameters such as *P* < 0.01, minimum term count of three, and enrichment factor >1.5.

### Analysis of tumor immune and immunosuppressive cell infiltration

To explore the potential correlation between MVP expression and tumor-infiltrating immune cells in various tumors, we conducted an analysis using the Tumor Immune Estimation Resource (TIMER2) 2.0 database [22]. This database offers a comprehensive evaluation and integration of immune cells based on RNA sequencing samples from the TCGA dataset, encompassing DC, CD4+ T cells, CD8+ T cells, B cells, neutrophils, and macrophages. Moreover, we assessed the relationship between MVP expression and three immunosuppressive cell types, namely myeloid-derived suppressor cells (MDSCs), cancer-associated fibroblasts (CAFs), and regulatory T (Treg) cells across multiple cancer types.

### Cell culture and transfection

Cell lines, including BEAS-2B, GES-1, A549, MCF-7, HepG2, H1650, AGS and U87, were cultured in DMEM or RPMI-1640 supplemented with 10% FBS, and incubated at 37°C in a humidified atmosphere containing 5% CO_2_. MVP knockdown was achieved by transfecting cells with MVP-specific siRNAs using Lipofectamine 2000 (Invitrogen, USA). Control cells were transfected with scrambled siRNA.

### RNA isolation and quantitative real-time PCR

The total RNA was extracted using TRIzol reagent (Invitrogen), and reverse transcription was performed using the Takara PrimeScript RT reagent kit to obtain complementary DNA (cDNA). The expression levels of MVP and GAPDH were measured by quantitative real-time PCR (RT-qPCR) utilizing the ABI 7900HT real-time PCR system (Applied Biosystems, USA). The primer sequences used in the PCR analysis are provided below: F: 5′-GGAGCGAGATCCCTCCAAAAT-3′ and R: 5′-GGCTGTTGTCATACTTCTCATGG-3′ for GAPDH; F: 5′-TACATCCGGCAGGACAATGAG-3′ and R: 5′-CTGTGCAGTAGTGACGTGGG-3′ for MVP.

### Western blot

Total protein was extracted from cancer cells using RIPA buffer containing protease and phosphatase inhibitors. The protein concentrations were measured using a BCA protein assay kit. Proteins were separated by SDS-PAGE and transferred onto PVDF membranes. The membranes were blocked with 5% non-fat milk and incubated with primary antibodies anti-MVP (sc-18701, 1:100, Santa Cruz, USA) and anti-GAPDH (#5174, 1:1000, Cell Signaling Technology, USA) overnight at 4°C, followed by incubation with secondary antibodies. The protein bands were visualized using an ECL detection system (Pierce, USA).

### Cell proliferation analysis

Cell viability was evaluated through various assays including the CCK-8 assay, 5-ethynyl-2’-deoxyuridine (EdU) assay, and colony formation assay. Initially, cells were seeded in 96-well plates and treated with either MVP siRNA or control siRNA. Following incubation for 24, 48, and 72 hours, CCK-8 reagent was added to each well and incubated at 37°C for 2 hours. Subsequently, the absorbance was measured at 450 nm using a microplate reader. In the EdU assay, cancer cells were seeded in 96-well plates and treated with MVP siRNA or control siRNA for 48 hours. Then, Edu reagent was introduced to each well and incubated at 37°C for 2 hours. The cells were fixed with 4% paraformaldehyde and stained with Hoechst 33342 according to the manufacturer’s instructions. The resulting cells were visualized under a fluorescence microscope. For the colony formation assay, cancer cells were seeded in 6-well plates and exposed to MVP siRNA or control siRNA for 48 hours. Following a 2-week incubation period, the cells were rinsed with PBS, fixed with 4% paraformaldehyde, and stained with crystal violet.

### Transwell assay

Cell migration and invasion were quantified utilizing the Transwell assay. In a concise summary, cancer cells were seeded in the upper chamber of Transwell plates, either with or without Matrigel. The lower chamber was subsequently filled with RPMI-1640 supplemented with 10% FBS. Following an incubation period of either 24 hours, the cells situated on the upper surface of the membrane were eliminated, while those on the lower surface were immobilized through fixation using 4% paraformaldehyde and stained with crystal violet. Subsequently, the cells were enumerated employing a microscope.

### IHC staining

IHC staining of paraffin-embedded tissues with antibodies anti-MVP (Santa Cruz, sc-18701, 1:100) was performed and scored according to standard procedures. The staining score was determined by two independent pathologists.

### Apoptosis analysis

To identify apoptotic cells, a PE Annexin V Apoptosis Detection Kit I (#559763, BD Biosciences, USA) was used according to the manufacturer’s protocol. The cells were analyzed by flow cytometry.

### Statistical analysis

All experiments were conducted using GraphPad Prism 8, and the results were expressed as means ± SD derived from at least three independent samples. To assess differences, an unpaired two-tailed *t*-test, as recommended for independent analysis, was employed. Statistical significance was determined by a *p*-value below 0.05. ^*^P < 0.05, ^**^P < 0.01, and ^***^P < 0.001 were used to denote significance levels.

### Data and materials availability

All data needed to evaluate the conclusions of this study are presented in this article.

## RESULTS

### MVP localization and expression profiles

**Figure 1:**
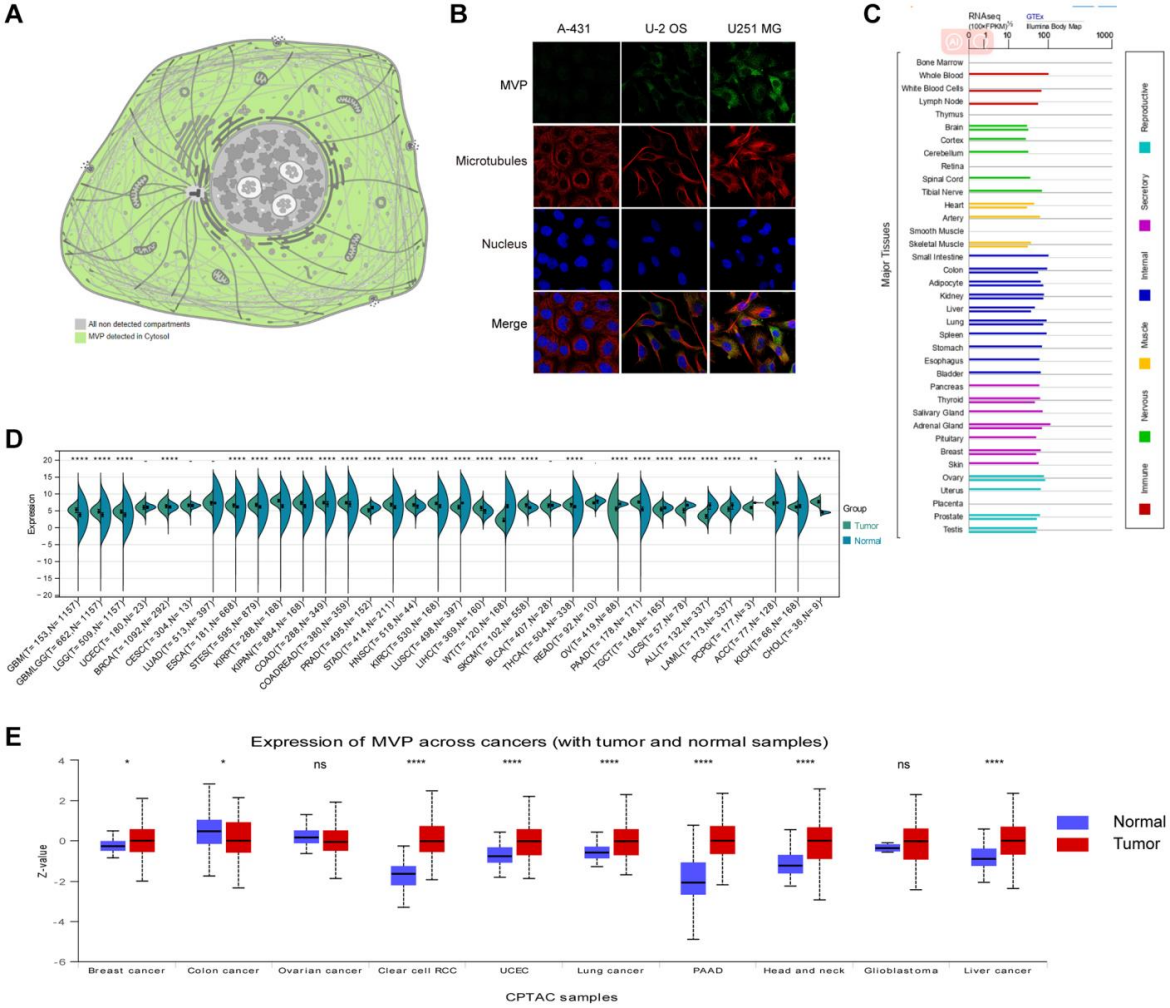
**MVP localization, functional chaperone, and expression profiles in normal tissues and cancer.** (**A**) MVP protein is localized in cytoplasm. (**B**) Immunofluorescence staining to detect the subcellular distribution of MVP in the nucleus, endoplasmic reticulum (ER) and microtubules of A-431 epidermoid carcinoma, U-2 osteosarcoma cells, and U251 glioblastoma. (**C**) Bar plot of MVP mRNA expression in various normal human tissues. (**D**) Expression levels of MVP mRNA in 34 different tumor types in the TCGA database via the SangerBox. (**E**) Expression levels of MVP protein in different tumors and corresponding normal tissues in the UALCAN portal. ^ns^*P* ≥ 0.05, ^*^P < 0.05, ^**^P < 0.01, ^***^P < 0.001, ^****^P < 0.0001.

MVP is primarily localized to the cytoplasm in human cells, as depicted in Figure 1A. To further characterize MVP’s intracellular localization, immunofluorescence assays to assess its distribution within the nuclei and microtubules of A-431, U-2 OS, and U251 MG cells. Our observations revealed that MVP colocalizes with cytoplasmic markers in cancer cells, providing strong evidence for subcellular localization of MVP in the cytoplasm (Figure 1B). Additionally, we investigated the expression of MVP messenger RNA (mRNA) across various normal human tissues, including immune, internal, neurological, secretory, muscular, and reproductive tissues (Figure 1C). Our findings underscored the widespread expression of MVP mRNA in these diverse tissue types, indicating its potential involvement in various cellular processes beyond cancer.

### Expressed levels of MVP in multiple human cancers

We conducted a comparative analysis to assess MVP expression in various types of cancer by comparing it between normal and tumor samples. Our findings indicate that among the 34 tumor types examined, 18 of them, including glioblastoma multiforme (GBM), glioblastoma (GBMLGG), and brain lower grade glioma (LGG), exhibited a significant upregulation of MVP mRNA levels compared to adjacent normal tissues (*P* < 0.05). Conversely, MVP expression was downregulated in 10 out of the 34 tumor types, including prostate cancer (PRAD), lung squamous cell carcinoma (LUSC), and Wilms tumor (WT), among others (Figure 1D). Furthermore, we utilized the CPTAC dataset to investigate MVP protein expression across 10 different cancer types. Our findings revealed that MVP expression was only decreased in colon cancer (COAD) and ovarian cancer (OV) when compared to neighboring non-cancerous tissues. In contrast, in several other cancer types, including breast cancer (BRCA), kidney clear cell carcinoma (KIRC), endometrial carcinoma (UCEA), lung cancer, pancreatic cancer (PAAD), head and neck cancer (HNSC), glioblastoma (GBM), and liver cancer (LIHC), MVP expression were significantly elevated compared to normal tissues (Figure 1E).

### Prognostic analysis of MVP in multiple human cancers

**Figure 2:**
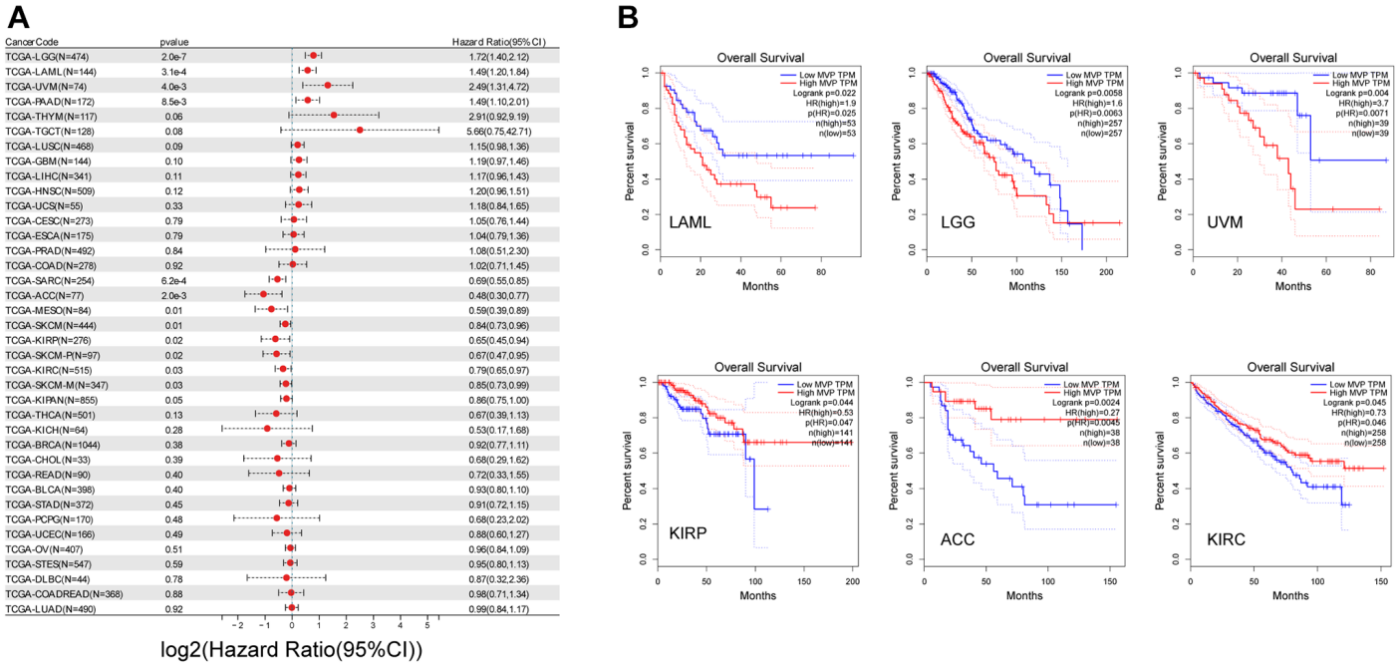
**MVP expression is closely related to the prognosis of cancers.** (**A**) Univariate Cox regression analysis using data obtained from the TCGA database to investigate the relationship between MVP expression levels and OS in different cancer types. (**B**) Kaplan-Meier curve of cumulative survival difference between TCGA cancer cohorts with high expression levels and low expression levels MVP. Only TCGA cancers with statistically significant differences between cohorts are presented.

In our analysis, we conducted univariate Cox regression analysis using data obtained from the TCGA and GTEx databases to examine the relationship between MVP expression levels and overall survival (OS) across different cancer types. The results revealed distinct prognostic implications, with high MVP expression associated with poorer prognosis in lower grade glioma and glioblastoma (GBM-LGG), acute myeloid leukemia (LAML), uveal melanoma (UVM), pancreatic adenocarcinoma (PAAD), and low expression associated with better prognosis in sarcoma (SARC), adrenocortical cancer (ACC), melanoma (SKCM), mesothelioma (MESO), kidney papillary cell carcinoma (KIRP), clear cell carcinoma of the kidney (KIRC), and pan-kidney cancer (KIPAN) (Figure 2A). Furthermore, using the TCGA database, we found that high MVP expression in LAML, LGG, and UVM tumors was associated with shorter OS, indicating a detrimental effect of MVP overexpression in these cancer types. Conversely, in KIRP, ACC, and clear cell carcinoma of the kidney (KIRC), overexpression of MVP was associated with longer OS, suggesting a potentially protective role of MVP in these specific cancer contexts (Figure 2B). These findings underscore the intricate and context-dependent nature of the role of MVP in tumorigenesis and development, with its functions exhibiting complete opposites in different tumor types. The internal mechanisms governing these diverse effects are likely extremely complex and warrant further investigation.

### DNA methylation analysis of MVP in pan-cancer

**Figure 3:**
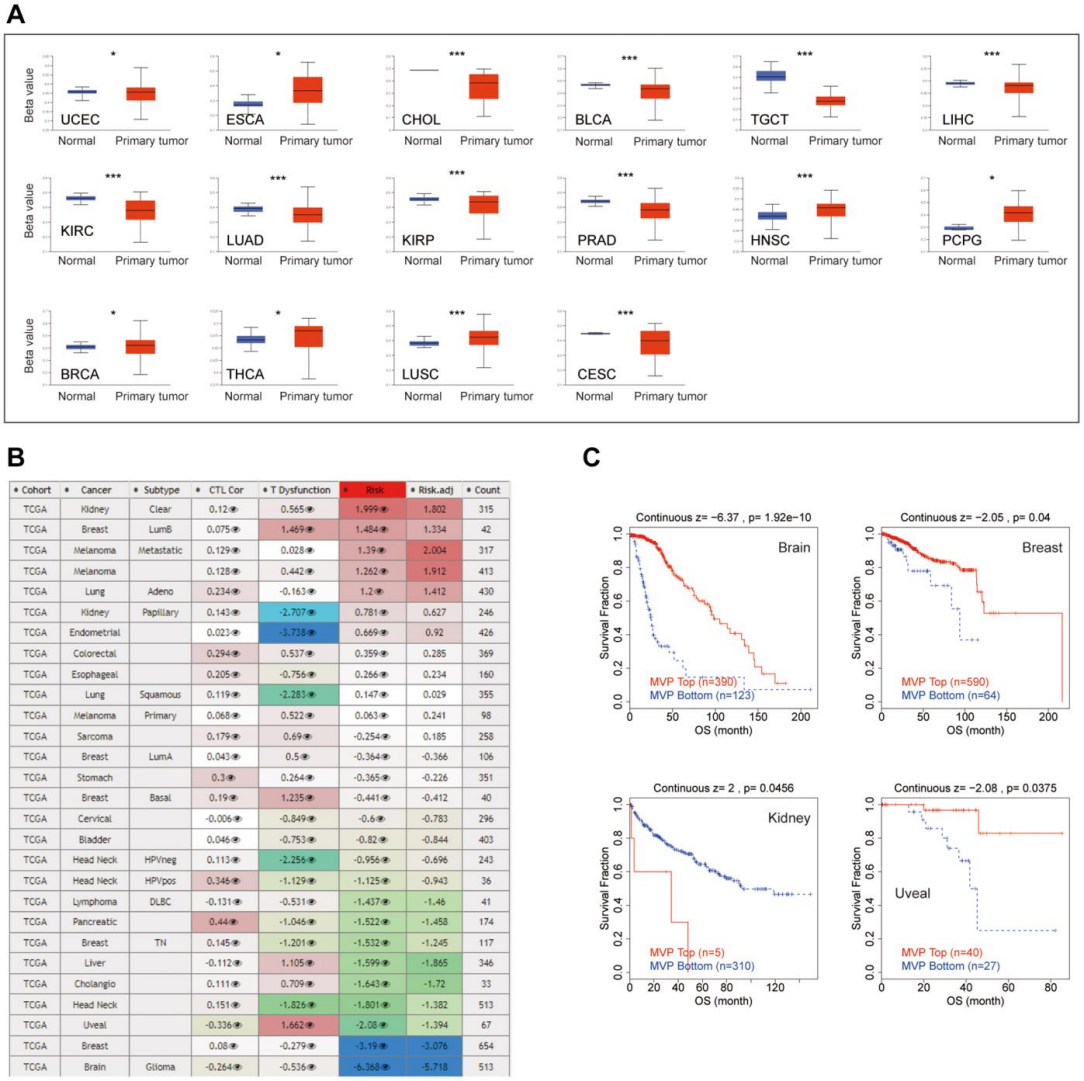
**Epigenetic methylation analysis.** (**A**) Boxplots illustrating differential MVP methylation levels between tumors and adjacent normal tissues in the TCGA database. (**B**) Heatmap demonstrating the impact of MVP methylation on cytotoxic T cell levels (CTL), dysfunctional T cell phenotype, and risk factors in the TCGA cancer cohort. (**C**) Kaplan-Meier curve displaying the disparity in OS between the TCGA cancer cohort with high methylation levels and the TCGA cancer cohort with low methylation levels of MVP. Only TCGA cancers with statistically significant differences between the cohorts are included.

The promoter methylation state analysis revealed hypermethylation of MVP in several cancer types, namely esophageal cancer (ESCA), HNSC, pheochromocytoma and paraganglioma (PCPG), BRCA, thyroid cancer (THCA) and LUSC. Conversely, MVP exhibited hypomethylation in bile duct cancer (CHOL), endometrioid cancer (UCEC), testicular cancer (TGCT), KIRC, lung adenocarcinoma (LUAD), PRAD, Cervical Cancer (CESC), and bladder cancer (BLCA) cancer types, as illustrated in Figure 3A. Furthermore, we examined the impact of MVP methylation status on cytotoxic T cell (CTL) activity, dysfunctional T cell phenotype, and various cancer risk factors, as depicted in Figure 3B. Our analysis demonstrated a positive correlation between hypomethylation of MVP and dysfunctional T cell phenotype, leading to reduced OS in brain, breast cancer, and uveal cohorts. Conversely, hypomethylation of MVP was associated with a favorable prognosis in kidney, as shown in Figure 3C. These results suggest that the epigenetic methylation levels of MVP in cancer patients are linked to dysfunctional T cell phenotypes, resulting in unfavorable outcomes for the brain, breast cancer, and uveal cohort, while improving survival in the kidney cohort.

### Analysis of MVP genetic alterations in different cancers

**Figure 4:**
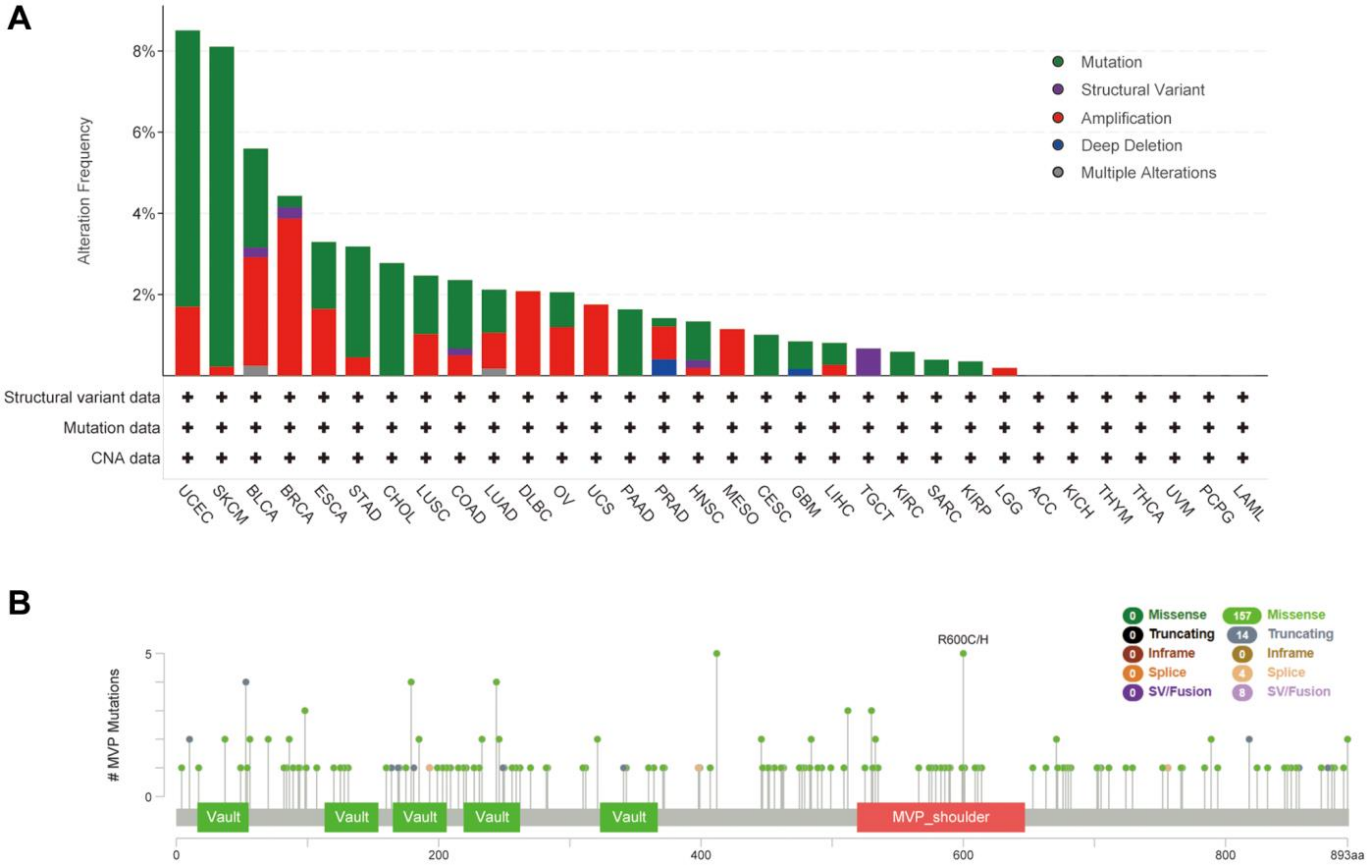
**Mutational characteristics of MVPs in various tumors.** The mutational profile of MVPs in tumors was analyzed utilizing the cBioPortal tool, the frequency of mutation types (**A**) and mutation sites (**B**) is presented.

Using the cBioPortal online database, we conducted an analysis of genetic alterations in MVP across various cancer types, as depicted in Figure 4A. The most prevalent genetic alteration in MVP was the “mutated” type, observed in nearly all TCGA cancer cases. Following this, the “amplified” type was identified, while multiple alterations were infrequent. Notably, the highest frequency of MVP alterations (>8%) was observed in UCEC and SKCM patients, primarily in the form of “mutation”. In BRAC samples, the prevailing alteration type was “amplification”. Furthermore, we investigated specific information regarding genetic alterations in MVP across different tumors. Our findings revealed that missense mutations were the predominant alteration type (Figure 4B), where R600C/H alterations were predominantly observed.

### MVP-related gene enrichment analysis

**Figure 5:**
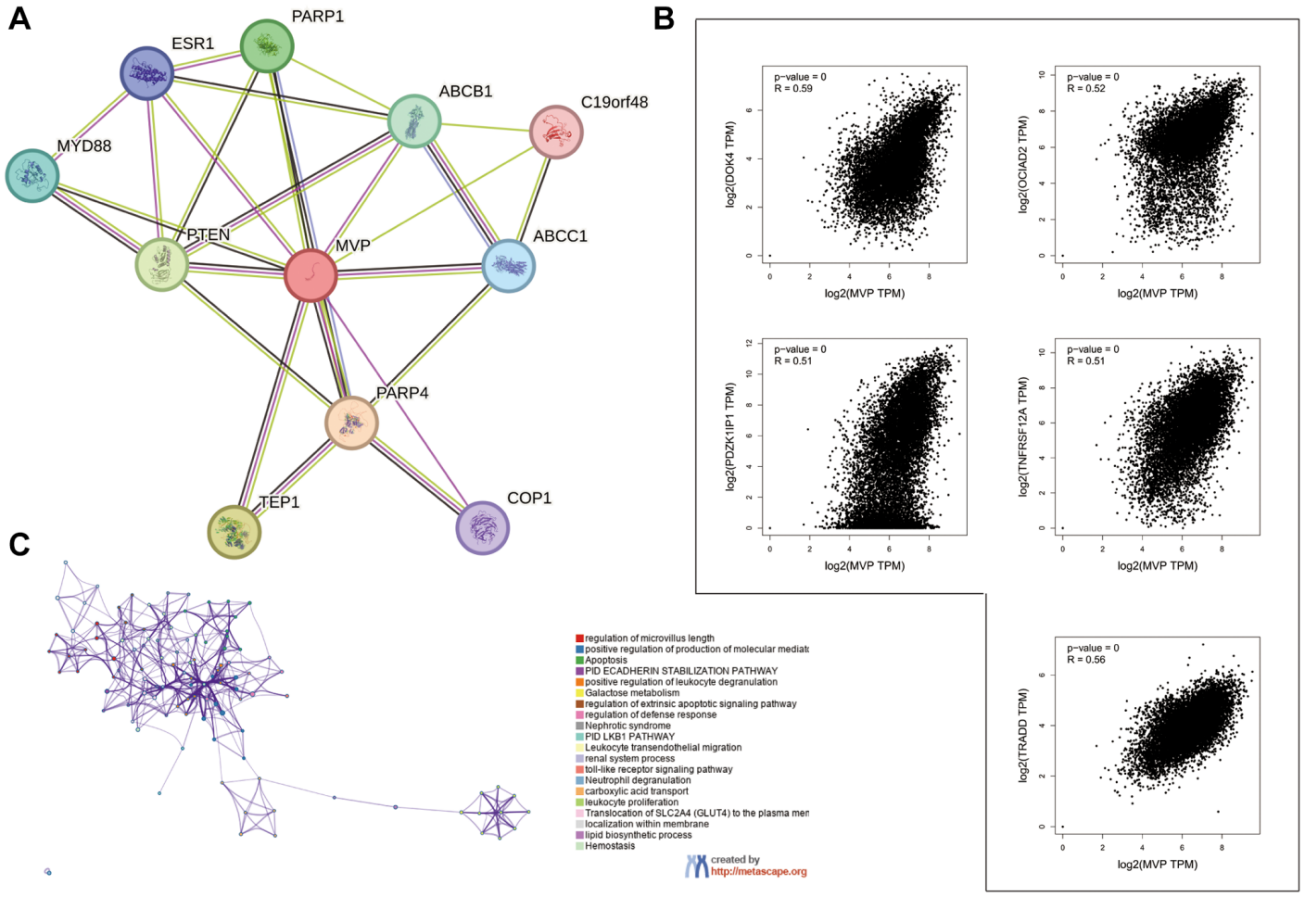
**Enrichment analysis of MVP-related genes in pan-cancers.** (**A**) MVP-binding proteins were identified using the STRING tool. (**B**) The top 5 genes associated with MVP in the TCGA project were obtained using the GEPIA2, and their expression correlation with MVP was analyzed. (**C**) Enriched terms with a similarity greater than 0.3 are depicted by connecting edges.

To elucidate the functional mechanism of MVP in carcinogenesis, we employed the STRING tool to identify genes that exhibited co-expression with MVP, as illustrated in Figure 5A. Among the various cancer types analyzed, the top five genes displaying a positive correlation with the MVP gene were DOK4, OCIAD2, PDZK1IP1, TNFRSF12A, and TRADD, as depicted in Figure 5B. Furthermore, gene pathway enrichment analysis revealed that the biological processes involving the top 100 MVP-associated genes were closely associated with apoptosis-related pathways, as shown in Figure 5C.

### MVP regulates tumor infiltration of immune cells in multiple human cancers

**Figure 6:**
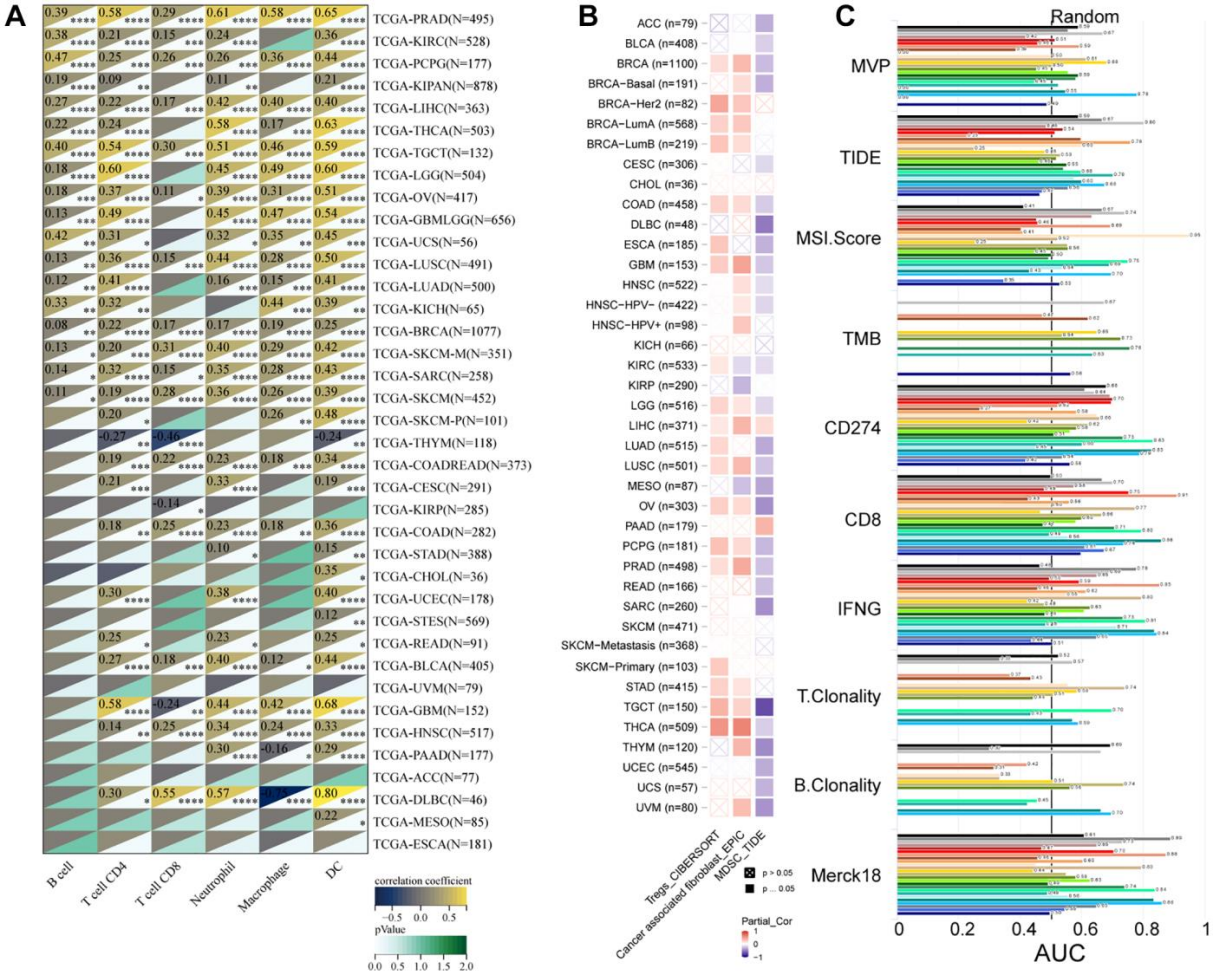
**Correlation of MVP expression with immune cell types and immunosuppressive cell types in TCGA cancer types.** (**A**) Heatmap displaying the correlation between MVP expression and six immune cell types in various TCGA cancer types. (**B**) Heatmap illustrating the correlation between MVP expression and three immunosuppressive cell types of infiltration in different TCGA cancer types. (**C**) Bar chart presenting the correlation of MVP with standardized cancer immune evasion biomarkers in the immune checkpoint blockade (ICB) subcohort. The biomarkers include CAFs (cancer-associated fibroblasts), MDSCs (myeloid-derived suppressor cells), and Tregs (regulatory T cells).

**Figure 7:**
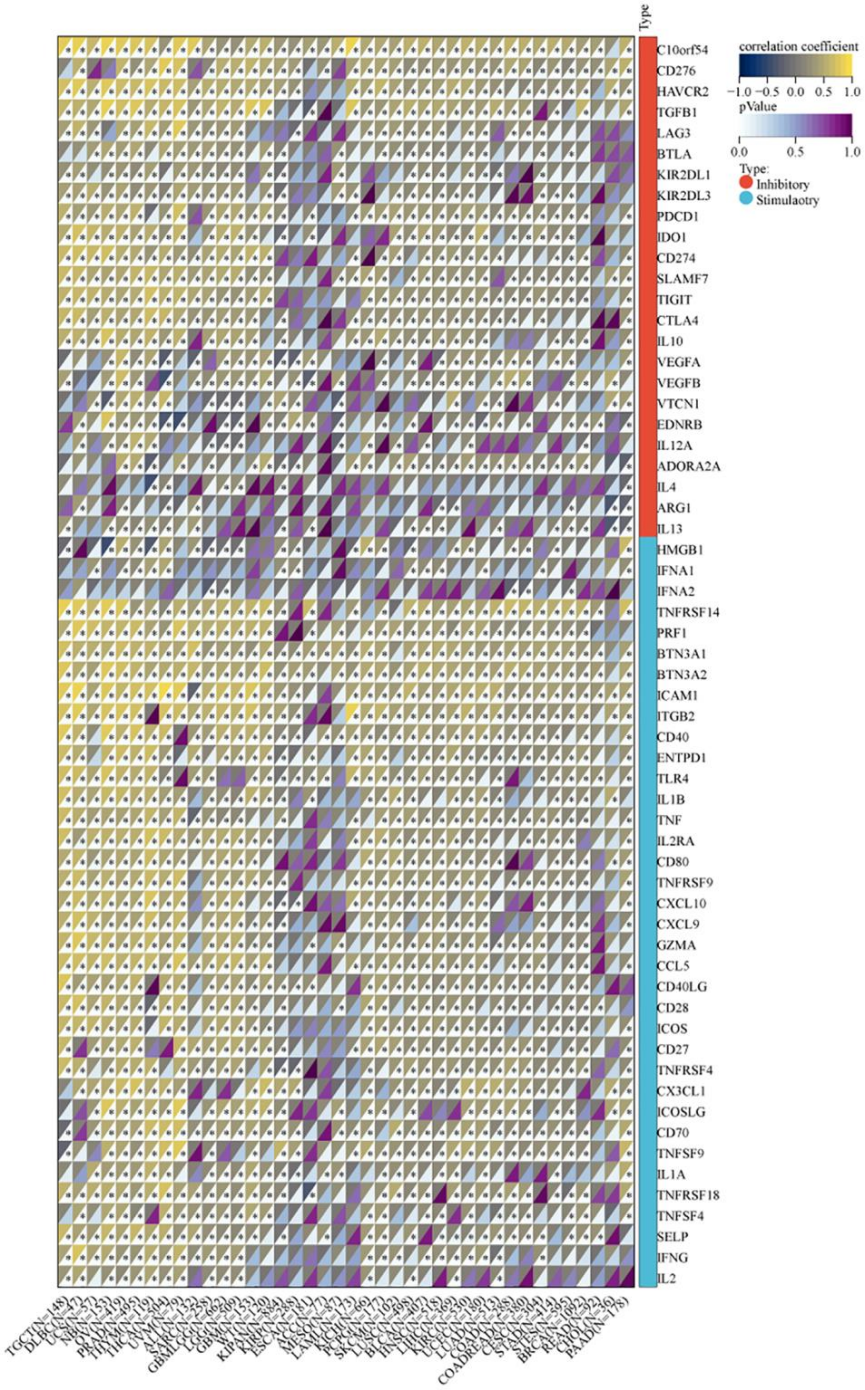
Correlation between MVP and two types of immune checkpoint pathway genes (inhibitory, stimulatory).

We utilized the SangerBox tool to investigate the association between MVP expression and infiltration by six immune cell types (B cells, CD8+ T cells, CD4+ T cells, macrophages, neutrophils, and dendritic cells) across 38 diverse TCGA cancer types. Our findings revealed significant positive correlations between MVP expression and immune cell infiltration in PRAD, PCPG, LIHC, TGCT, OV, LUSC, BRCA, SKCM, SARC, and PCPG. Conversely, only in thymoma (THYM) and KIRP, a significant negative correlation was observed between MVP expression and invasion by tumor-infiltrating immune cells (Figure 6A). These results indicate a positive correlation between MVP expression levels and tumor immune responsiveness in most tumor types. Additionally, we examined the correlation between MVP expression levels and infiltration by immunosuppressive cells (MDSC, CAF, and Treg cells). The results showed that MVP expression was negatively correlated with MDSC infiltration in most tumors. In contrast, MVP expression exhibited a positive correlation with CAF and Treg cell infiltration in BRCA, COAD, GBM, TGCT, thyroid carcinoma (THCA), THYM, and other tumors (Figure 6B). Furthermore, we compared the predictive ability of MVP with standardized biomarkers in relation to response outcomes and OS in ICB sub-cohorts. Remarkably, MVP alone exhibited an area under the receiver operating characteristic curve (AUC) of ≥0.5 in 15 out of the 25 ICB sub-cohorts (Figure 6C). Compared to the MIS score, TMB, T. Clonality, and B. Clonality, MVP demonstrated superior predictive value. Additionally, we conducted an analysis of the relationship between MVP and 60 immune checkpoint modulators (divided into two classes: Inhibitory and Stimulatory) across 40 cancer types. Our findings indicate a predominant positive correlation between MVP expression and immunomodulators in the majority of tumors (Figure 7).

### MVP accelerated the proliferation and migration of A549 and AGS *in vitro*

**Figure 8:**
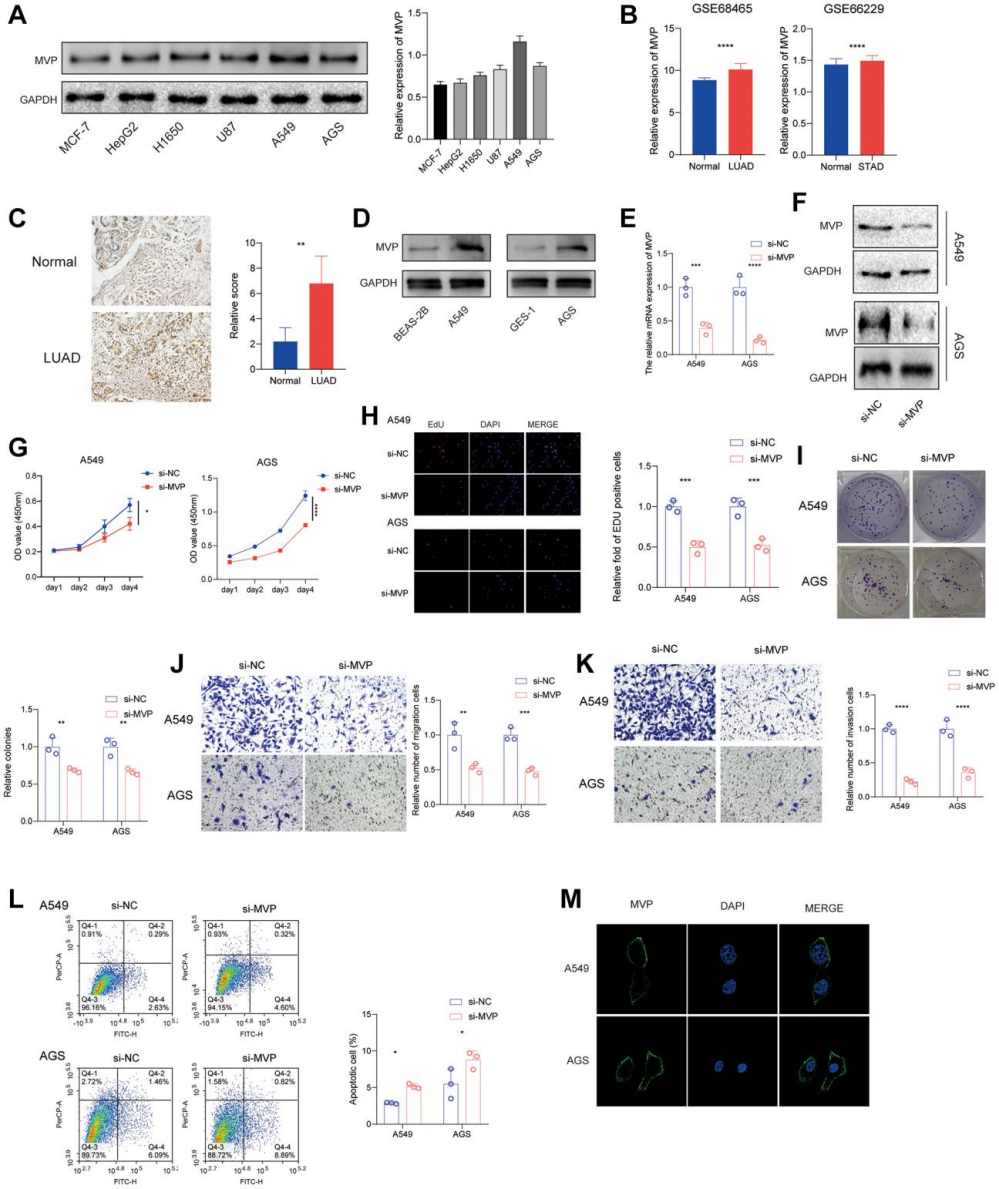
**MVP knockdown effects in cancer cells.** (**A**) The protein expression level of MVP in each cell line was tested by western blot. (**B**) Analysis of the GEO database showed that MVP was highly expressed in LUAD and STAD compared to adjacent tissues. (**C**) Immunohistochemical staining showed that MVP was highly expressed in LUAD compared with adjacent tissues. (**D**) Western blot assay confirmed that MVP expression was higher in A549 and AGS cells than in their corresponding normal epithelial cells. (**E**, **F**) RT-qPCR and western blot confirming MVP knockdown efficiency in A549 and AGS. (**G**) CCK-8 assay measuring cell proliferation in MVP-knockdown and control cells. (**H**) EdU assay measuring cell proliferation in MVP-knockdown and control cells. (**I**) Colony formation assay showed that MVP knockdown inhibits the colony formation ability of cells. (**J**, **K**) Transwell migration and invasion assays showing decreased migration and invasion of MVP-knockdown cells. **(L**) Cell apoptosis was monitored by flow cytometry. (**M**) Immunofluorescence assay showed that MVP was mainly distributed in the cell membrane and cytoplasm of A549 and AGS cells. ^*^P < 0.05, ^**^P < 0.01, ^***^P < 0.001, ^****^P < 0.0001.

In order to investigate the functional role of MVP in cancers, we assessed the protein expression levels of MVP in various tumor cells. The results revealed the highest expression level in A549 and AGS (Figure 8A). Furthermore, the GEO database showed that MVP was highly expressed in LUAD and STAD compared with adjacent tissues (Figure 8B). Immunohistochemical data showed the same results in lung cancer (Figure 8C). Western blot assay confirmed that MVP expression was higher in A549 and AGS cells than in their corresponding normal epithelial cells (Figure 8D). Consequently, we conducted *in vitro* experiments utilizing A549 and AGS cells. Initially, we confirmed the efficacy of siRNAs targeting MVP through RT-qPCR and western blot analysis, which demonstrated a significant reduction in MVP expression in A549 and AGS cells compared to the control group (Figure 8E, 8F). Knocking down MVP inhibited cell proliferation, as shown in CCK-8 and EdU assays (Figure 8G, 8H). MVP knockdown also reduced colony formation, indicating its role in promoting cancer cell growth (Figure 8I). Additionally, MVP knockdown decreased cell migration and invasion, suggesting its involvement in these processes (Figure 8J, 8K). Furthermore, MVP knockdown promote apoptosis (Figure 8L). Immunofluorescence assay showed that MVP was mainly distributed in the cell membrane and cytoplasm of A549 and AGS cells, but not in the nucleus (Figure 8M). Overall, our results demonstrate that MVP acts as a tumor promoter in cancers by enhancing cell proliferation, migration, invasion and attenuated apoptosis.

## DISCUSSION

MVP, a pivotal component of cellular ribonucleoprotein particles, plays a crucial role in regulating various cellular processes, including cell differentiation, nucleoplasmic transport, signal transduction, and immune response [23, 24]. Despite its significance, our comprehensive understanding of the involvement of MVP in tumorigenesis and development remains limited. To bridge this knowledge gap, we conducted a comprehensive bioinformatics analysis using patient data from multiple databases to explore the oncogenic effects of MVP. Our investigation revealed that MVP is widely expressed in various human tissues, with upregulated mRNA levels observed in most tumors compared to adjacent normal tissues. However, it’s worth noting that there are discrepancies among the results from different databases, underscoring the need for further research to comprehensively analyze the functions and characteristics of MVP. Furthermore, our analysis uncovered a correlation between MVP overexpression and poor prognosis in certain cancers, such as LAML, LGG, and UVM. Conversely, in KIRP, ACC, and KIRC, high MVP expression was positively associated with better OS. These findings align with previous studies that have linked MVP overexpression to unfavorable prognosis and reduced chemotherapy sensitivity in multiple myeloma, prostate cancer, acute myelogenous leukemia, and non-small cell carcinoma. Our analysis also indicated differential methylation patterns of MVP in various cancer types, with hypomethylation observed in some, such as CHOL, KIRC, TGCT, LIHC, LUAD, KIRP, PRAD, CESC, and BLCA, and hypermethylation observed in others, including UCEC, ESCA, HNSC, PCPG, THCA, and LUSC. Investigating the mechanisms behind these epigenetic changes and their impact on MVP expression deserves further study.

The primary objective of this study is to investigate the complex network of interactions among various biological factors within the context of a specific cancer type. For instance, in patients with lower grade glioma (LGG), we have observed a strong correlation between the expression of MVP, patient prognosis, DNA methylation, and the immune cell profile. Our findings indicate that both MVP mRNA and protein exhibit significantly higher levels in LGG tissues, which are closely associated with patient prognosis. Additionally, we have identified a link between hypermethylation of MVP and poorer prognosis. However, the precise mechanisms underlying epigenetic changes affecting MVP levels warrant further investigation. The immune microenvironment within the tumor ecosystem plays a pivotal role in cancer progression. The results have shown a positive correlation between MVP expression and the presence of dendritic cells (DC) and macrophages in LGG. This integrated analysis sets the stage for a more profound comprehension of the molecular mechanisms driving cancer progression and may provide insights into personalized therapeutic strategies tailored to each patient’s unique biological landscape.

While our study underscores the potential of MVP as a prognostic biomarker across multiple malignancies, the question of how MVP’s non-specific expression pattern can be harnessed for cancer subtype identification is both pertinent and intriguing. The non-specific nature of expression of MVP indeed poses challenges in utilizing it as a standalone diagnostic marker for a specific malignancy. One promising avenue for further investigation lies in the integration of multiple molecular features. By combining MVP expression data with other relevant molecular markers, such as genetic alterations, epigenetic modifications, and immune cell profiles, it may be possible to derive a composite score or signature specific to certain malignancies. Moreover, leveraging advanced machine learning and artificial intelligence algorithms for feature selection and pattern recognition could aid in the development of robust classification models. These models could potentially differentiate between cancer subtypes based on the intricate interplay of molecular features, providing clinicians with valuable insights for tailored treatment strategies. While non-specific expression pattern of MVP hints at its utility as a broad prognostic biomarker, the path to utilizing it for precise cancer subtype identification involves further exploration of combined molecular signatures and advanced computational approaches.

In summary, this study has centered on exploring the potential role of MVP as a prognostic and immunological biomarker in a diverse range of cancer types. Leveraging extensive transcriptomic and clinical data from publicly available databases, our investigation has yielded critical insights into the significance of MVP in the realm of cancer biology. We have conducted experimental validation in cancer cell lines to validate the expression and functional role of MVP. This experimental component fortifies the robustness of our findings and underscores the relevance of MVP in the realm of cancer biology.
